# *Nitrosomonas europaea* MazF Specifically Recognises the UGG Motif and Promotes Selective RNA Degradation

**DOI:** 10.3389/fmicb.2018.02386

**Published:** 2018-10-08

**Authors:** Tatsuki Miyamoto, Akiko Yokota, Yuri Ota, Masako Tsuruga, Rie Aoi, Satoshi Tsuneda, Naohiro Noda

**Affiliations:** ^1^Department of Life Science and Medical Bioscience, Waseda University, Tokyo, Japan; ^2^Biomedical Research Institute, National Institute of Advanced Industrial Science and Technology (AIST), Ibaraki, Japan

**Keywords:** toxin-antitoxin system, MazEF, sequence-specificity, RNase, *Nitrosomonas europaea*, ammonia oxidation, carbon fixation

## Abstract

Toxin-antitoxin (TA) systems are implicated in prokaryotic stress adaptation. Previously, bioinformatics analysis predicted that such systems are abundant in some slowly growing chemolithotrophs; e.g., *Nitrosomonas europaea*. Nevertheless, the molecular functions of these stress-response modules remain largely unclear, limiting insight regarding their physiological roles. Herein, we show that one of the putative MazF family members, encoded at the ALW85_RS04820 locus, constitutes a functional toxin that engenders a TA pair with its cognate MazE antitoxin. The coordinate application of a specialised RNA-Seq and a fluorescence quenching technique clarified that a unique triplet, UGG, serves as the determinant for MazF cleavage. Notably, statistical analysis predicted that two transcripts, which are unique in the autotroph, comprise the prime targets of the MazF endoribonuclease: hydroxylamine dehydrogenase (*hao*), which is essential for ammonia oxidation, and a large subunit of ribulose 1,5-bisphosphate carboxylase/oxygenase (*rbcL*), which plays an important role in carbon assimilation. Given that *N. europaea* obtains energy and reductants via ammonia oxidation and the carbon for its growth from carbon dioxide, the chemolithotroph might use the MazF endoribonuclease to modulate its translation profile and subsequent biochemical reactions.

## Introduction

Toxin-antitoxin (TA) systems comprise small genetic modules that modulate microbial cell fates in stressful environments. During periods of low or no stress, antitoxins potently inactivate toxins, thus allowing microbes to grow normally. Under environmental stress, however, antitoxins are preferentially degraded, resulting in toxin activation and subsequent microbial growth arrest (Schuster and Bertram, 2013). Currently, these systems are grouped into six distinct classes depending on antitoxin features (Page and Peti, 2016; Hall et al., 2017). In type I and type III, the antitoxins comprise RNAs that mask toxin activities by repressing toxin translation or directly binding to corresponding toxins, respectively (Brantl, 2012; Goeders et al., 2016). In other TA systems, the antitoxins consist of proteins that counteract the toxins by forming a TA protein complex (type II) (Gerdes et al., 2005), functioning as an antagonist of the toxin (type IV) (Masuda et al., 2012a,b), inhibiting translation by destabilising the toxin mRNA (type V) (Wang et al., 2012), or serving as an adaptor molecule to introduce the toxin to cellular protease (type VI) (Aakre et al., 2013). Among these, type II systems have been rigorously investigated and are known to be ubiquitous and abundant in free-living microbes, especially in some chemolithotrophic prokaryotes (Pandey and Gerdes, 2005).

*Nitrosomonas europaea*, which is predicted to carry more than 50 type II TA pairs, is one such chemolithotroph (Shao et al., 2011; Xie et al., 2017). *N. europaea* obtains energy and reductants by oxidising ammonia to nitrite (Arp et al., 2002; Arp and Stein, 2003), inhabiting wastewater treatment and sediments where ammonia may be in abundant supply (Koops et al., 2006). As the oxidation process is important in terms of industrial, agricultural, and environmental nitrogen cycles (Bothe et al., 2000), the physiology, biochemistry, and molecular biology of this bacteria in varied environments has been investigated. It has been well documented that *N. europaea* is susceptible to numbers of environmental factors such as temperature, pH, nitrite and ammonia concentrations, heavy metals, and organic and inorganic compounds (Groeneweg et al., 1994; Stein and Arp, 1998; Koops et al., 2006; Wei et al., 2006a,b; Park and Ely, 2008a,b; Radniecki et al., 2009; Yu and Chandran, 2010; Pellitteri-Hahn et al., 2011). Considering that *N. europaea* harbours a very high number of putative TA pairs (Pandey and Gerdes, 2005), this bacterium might regulate cellular activities using type II toxins.

Among the putative *N. europaea* toxins, a great majority are predicted to catalyse cellular RNA decay (Pandey and Gerdes, 2005; Shao et al., 2011; Xie et al., 2017); *Escherichia coli* MazF, a sequence-specific toxin endoribonuclease (Zhang et al., 2003), provides a helpful basis to better understand these endoribonucleolytic toxins in *N. europaea*. In the absence of stresses, MazF-mediated RNA cleavage is neutralised by its cognate antitoxin MazE (Zhang et al., 2003). Environmental stresses such as starvation, high temperature, and oxidative stress (Hazan et al., 2004), however, trigger MazE degradation via the ClpAP protease (Aizenman et al., 1996), activating the MazF endoribonuclease. *E. coli* MazF then comprehensively alters the translation profile by cleaving transcripts at ACA sites (Amitai et al., 2009; Sauert et al., 2016). MazF homologues are widely distributed in the prokaryotic domain (Chopra et al., 2013), with a hallmark of these enzymes being their sequence-specificities (Masuda and Inouye, 2017; Schifano and Woychik, 2017). Typically, MazF toxins recognise three to seven specific-sequences (i.e., ACA, UACAU, and UUACUCA), suggesting that the biological roles of MazF homologues are diverse and differ across microbial species (Zhu et al., 2006, 2008, 2009; Park et al., 2011; Rothenbacher et al., 2012; Schifano et al., 2013, 2014; Schuster et al., 2013; Verma and Bhatnagar, 2014; Miyamoto et al., 2016b, 2017; Li et al., 2017).

Notably, five *mazEF* loci are predicted in the *N. europaea* genome, whereas only one is found in the majority of prokaryotes (Pandey and Gerdes, 2005; Chopra et al., 2013). The cleavage-specificities of these endoribonucleases are less well studied and largely unknown, except for one located at the ALW85_RS06130 locus (previously designated as NE1181) (Miyamoto et al., 2016b); therefore, detailed analysis is required to interpret their physiological functions. In the present work, we focused our study on the predicted MazF endoribonuclease located at ALW85_RS04820 (hereafter MazFne1; Pandey and Gerdes, 2005) and revealed that it codes for a functional toxin endoribonuclease. Our study suggests that MazFne1 may serve as a crucial regulator of translation and cellular activities.

## Materials and Methods

### Plasmids and Oligonucleotides

The pET24a expression vectors encoding *mazE*ne1 and pET24a encoding *mazF*ne1 were purchased from GenScript Japan (Tokyo, Japan). The sequences of both genes were optimised for recombinant protein expression in *E. coli*. PCR primers were purchased from Tsukuba Oligo Service (Ibaraki, Japan). All fluorescently labelled oligonucleotides were purchased from Japan Bio Services (Saitama, Japan).

### Reverse Transcription (RT)-PCR

*Nitrosomonas europaea* ATCC 19718 was obtained from the Biological Resource Center (NBRC), National Institute of Technology and Evaluation (NITE), Japan. *N. europaea* was cultivated in the dark at 28°C. HEPES medium 829 was used as the growth medium, as described in the NBRC manual. Cells were harvested by centrifugation at 2900 g. The cell pellet was transferred to a 2 mL screw-cap tube containing glass beads (0.1 mm in diameter) and disrupted in TRI-Reagent (Zymo Research, Orange, CA, United States). Total RNA was extracted using the Direct-zol^TM^ RNA MiniPrep Kit (Zymo Research) according to the manufacturer’s protocol. Extracted RNA was then incubated with 4U of TURBO DNase (Thermo Fisher Scientific, Waltham, MA, United States) in the TURBO DNase Buffer at 37°C for 1 h and purified with RNA Clean and Concentrator^TM^-5 (Zymo Research). Resultant RNA was incubated at 65°C for 5 min and cDNA was synthesised using the SuperScript VILO cDNA Synthesis Kit (Thermo Fisher Scientific) according to the manufacturer’s protocol. PCR was carried out with Q5 High-Fidelity DNA Polymerase (New England Biolabs, Ipswich, MA, United States) using the primers listed in **Supplementary Table 1**. PCR conditions were as follows: an initial denaturation at 98°C for 1 min, followed by 30 cycles of amplification (denaturation at 98°C for 10 s, annealing at 65°C for 15 s, and extension at 72°C for 20 s), and a final extension for 2 min at 72°C. The samples were separated on a 2% agarose gel and visualized by GelRed^TM^ Nucleic Acid Gel Stain (Biotium, Inc., Fremont, CA, United States).

### Toxicity Assay

*E. coli* strain BL21 (DE3) cells (BioDynamics Laboratory Inc., Tokyo, Japan) were transformed with pET24a-*mazF*ne1. These cells were pre-cultivated overnight in LB medium supplemented with 50 μg/mL kanamycin at 37°C. Cells were diluted 1:1000 in 10 mL of LB medium containing 50 μg/mL kanamycin. MazFne1 was induced by the addition of 1 mM isopropyl β-D-1-thiogalactopyranoside (IPTG) when the OD_600_ reached approximately 0.1. OD_600_ was measured at every 30 min.

### Expression and Purification of MazEne1

Recombinant MazEne1 was obtained as outlined in our previous study (Miyamoto et al., 2016b) with slight modifications. *E. coli* strain BL21 (DE3) cells (BioDynamics Laboratory Inc.) were transformed with pET24a-*mazE*ne1. These cells were pre-cultivated overnight in LB medium supplemented with 20 μg/mL kanamycin at 37°C. Subsequently, they were inoculated into 1 L of LB medium containing 20 μg/mL kanamycin. MazEne1 was induced by the addition of 1 mM IPTG when the OD_600_ reached approximately 0.6. After 3.5 h of incubation, the cells were harvested by centrifugation at 9200 g and stored at -80°C until further use. *E. coli* cells containing MazE were thawed on ice and resuspended in 18 mL of binding buffer (20 mM sodium phosphate buffer (pH 8.0), 300 mM NaCl, 40 mM imidazole, and 5 mM 2-mercaptoethanol). Subsequently, these cells were lysed by sonication and collected by centrifugation at 4400 g. The supernatant was filtered through a 0.45 μm membrane (Millex, Darmstadt, Germany), and applied to an equilibrated 1 mL His-Trap FF crude column (GE Healthcare, Little Chalfont, United Kingdom). The column was washed with 40 column volumes of binding buffer using AKTA pure 25 (GE Healthcare). Histidine-tagged MazE was selectively eluted with the elution buffer (20 mM sodium phosphate buffer (pH 8.0), 300 mM NaCl, 500 mM imidazole, and 5 mM 2-mercaptoethanol) using the following program: flow rate, 1 mL/min; linear elution gradient, 20 column volumes; fraction size, 0.5 mL. The 18th fraction from the beginning of the elution program was used for further experiments. The molecular weight and purity were confirmed by sodium dodecyl sulfate polyacrylamide gel electrophoresis (SDS–PAGE). Protein concentration was determined using the Bio-Rad Protein Assay (Bio-Rad, Hercules, CA, United States).

### Expression and Purification of MazFne1

Recombinant MazFne1 was expressed and purified as described previously with minor modifications (Miyamoto et al., 2016b). *E. coli* strain BL21 (DE3) cells (BioDynamics Laboratory Inc.) was transformed with pET24a-*mazF*ne1. The transformant was cultivated overnight in LB medium supplemented with 20 μg/mL kanamycin at 37°C and inoculated into 1 L LB medium containing 20 μg/mL kanamycin. MazF was induced by the addition of 1 mM IPTG when the OD_600_ reached approximately 4.0. After 3.5 h of incubation, the cells were harvested by centrifugation at 9200 g and stored at -80°C. The cells containing MazFne1 were thawed on ice and resuspended in 18 mL of binding buffer (20 mM sodium phosphate buffer (pH 8.0), 300 mM NaCl, 40 mM imidazole, and 5 mM 2-mercaptoethanol). The cells were lysed by sonication and collected by centrifuging at 4400 g for 15 min. The supernatant was then filtered through a 0.45 μm membrane (Millex). After equilibrating a 1 mL His-Trap FF crude column (GE Healthcare), the supernatant was applied to the column and washed with 55 column volumes of binding buffer using AKTA pure 25 (GE Healthcare). Hexa-histidine tagged MazFne1 was selectively eluted using the following program: flow rate, 1 mL/min; linear elution gradient, 20 column volumes; fraction size, 0.5 mL. The elution buffer contained 20 mM sodium phosphate buffer (pH 8.0), 300 mM NaCl, 500 mM imidazole, and 5 mM 2-mercaptoethanol. The 17th and 18th fractions from the beginning of the elution program were used for further experiments. The molecular weight and purity were confirmed using SDS–PAGE. Protein concentration was determined using a Bio-Rad Protein Assay (Bio-Rad).

### Enzymatic Activity of MazF and MazE

Synthetic RNA constructs were prepared as described in our previous study (Miyamoto et al., 2016a). RNA 500-2 (100 ng) was incubated with 1, 3, or 10 pmol of MazF at 37°C for 90 min in MazF reaction buffer [20 mM Tris–HCl (pH 8.0), 1 mM dithiothreitol, 0.01% Triton X-100, and 4 U of recombinant RNase inhibitor (TaKaRa, Shiga, Japan)] in a 50 μL reaction volume. As a control reaction, 10 pmol of MazF was pre-incubated with 50 pmol of MazE at 25°C for 10 min, and this mixture was incubated with 100 ng of RNA 500-2 at 37°C for 90 min in MazF reaction buffer. These RNAs were purified with RNA Clean and Concentrator^TM^-5 (Zymo Research). Gel loading buffer II (Ambion, Austin, TX, United States) was added to each sample followed by incubation at 95°C for 5 min. Samples were separated on a 10% polyacrylamide gel containing 7 M urea. The RNA was stained using SYBR Gold (Life Technologies) and detected using a Typhoon 9210 imager (GE Healthcare).

### Cleavage Sequence Identification

The cleavage sequence was identified using the protocols described in our previous study (Miyamoto et al., 2016a, 2017). Briefly, 0.625 pmol of eight RNA mixtures (500-2, 1000-1, 1000-2, 1000-3, 1000-4, 1000-5, 1500-1, and 2000-1) were incubated with 50 ng of MazFne1 at 37°C for 30 min in MazF reaction buffer. Phosphorylation, barcode ligation, and sequencing library construction were performed as described previously (Miyamoto et al., 2016a, 2017). Sequencing was performed using the MiSeq platform with the MiSeq 500 cycles reagent kit v2 (Illumina, San Diego, CA, United States) according to the manufacturer’s protocol. Sequence data were analysed using CLC Genomics 7.5.1 (Qiagen, Venlo, The Netherlands). The parameters described in our previous study (Miyamoto et al., 2016a, 2017) were used for the analysis. We added pseudo-counts to the nucleotide positions whose coverages were 0, and calculated the relative coverage increase, the value defined as the coverage at the (*n* + 1)th position divided by the coverage at the *n*th position. Nucleotide positions with coverage less than 100 were excluded from analysis. Of these nucleotide positions, those showing the overall top 50 relative coverage increases were selected from all references. The sequences five-base pairs upstream and downstream of these positions were extracted and aligned using WebLogo (Crooks et al., 2004). These sequence data were submitted to the DDBJ database under the accession number DRA006546.

### Fluorometric Assay

The fluorometric assay was carried out as outlined in our previous study (Miyamoto et al., 2016a,b). Briefly, 0.7 pmol of MazFne1 or 100 ng of RNase A was incubated with 20 pmol of fluorescently labelled oligonucleotides in MazF reaction buffer in a total volume of 20 μL. As a control reaction, 0.7 pmol of MazFne1 was pre-incubated with 3.5 pmol of MazEne1 at 25°C for 10 min, and this mixture was incubated with 20 pmol of fluorescently labelled oligonucleotides in MazF reaction buffer in a total volume of 20 μL. All reactions were conducted at 37°C in triplicate and fluorescence intensity was recorded every 1 min using a Light Cycler 480 system (Roche, Basel, Switzerland) with 483 nm excitation and 533 nm detection filters.

### Analysis of UGG Frequency in *N. europaea* Coding Sequences

Statistical analysis was performed as outlined in a previous study (Zhu et al., 2009). Protein-coding sequences of *N. europaea* were retrieved from the NCBI database. The CDS data as of February 10, 2018 were used for the analysis. The following parameters were taken into account: (i) *p* is the probability of TGG appearing in a *N. europaea* gene and represents the product of (percentage of U) multiplied by (percentage of G)^2^; (ii) *L* represents the length of each coding sequence (CDS); (iii) *E* is the expected number of TGG motifs in a CDS and is calculated as *p*(*L* – 2); (iv) *K* is the actual number of TGG sequence in a CDS; and (v) *P* represents the probability of each CDS containing TGG motifs at least *K* times. *P* is given by:

$$P=\sum_{i=k}^{L-2}p^{i}{\left(1-p\right)}^{L-2-i}\frac{(L-2)!}{i!(L-2-i)!}$$

### Accession Numbers

The GenBank accession numbers are as follows: artificially designed RNAs: 500-2 (AB610940), 1000-1 (AB610944), 1000-2 (AB610945), 1000-3 (AB610946), 1000-4 (AB610947), 1000-5 (AB610948), 1500-1 (AB610949), and 2000-1 (AB610950); *N. europaea* genome (NC_004757); MazEne1 (WP_041356592); MazFne1 (WP_011111532).

## Results

### MazFne1 Constitutes a Toxic Protein That Is Co-transcribed With Its Upstream Gene

Although the putative ORF existing immediately upstream of the *mazF* gene, encoded at ALW85_RS04820 (originally annotated as NE0921; *mazF*ne1), was not initially annotated (Chain et al., 2003), bioinformatics analysis predicted that it codes for the MazE counterpart (MazEne1) (**Supplementary Figure 1A**) (Pandey and Gerdes, 2005), with MazEne1 and MazFne1 exhibiting 20.7 and 18.7% amino acid sequence identities to those of *E. coli*, respectively (**Supplementary Figure 1B**). Moreover, the *mazEF*ne1 genes are organised in a manner fully consistent with the type II TA system (Sevin and Barloy-Hubler, 2007), with an 11 bp overlap between *mazE*ne1 and *mazF*ne1 (**Supplementary Figure 2A**).

A common feature of type II TA systems is their operon structure (Gerdes et al., 2005). To investigate whether the putative *mazEF* genes are co-transcribed in *N. europaea*, we carried out reverse transcription (RT)-PCR using three different primer sets (**Supplementary Figure 2A**, upper panel): one for the *mazE*ne1 gene (primers 1 and 2), another for *mazF*ne1 (primers 3 and 4), and the third for *mazEF*ne1 (primers 1 and 4). In all cases, we observed amplification only in the presence of reverse transcriptase (**Supplementary Figure 2A**, lower panel), demonstrating that *mazE*ne1 and *mazF*ne1 are co-transcribed.

Another key feature of TA pairs is the effect of toxin expression on bacterial growth. To briefly investigate whether MazFne1 constitutes a toxin, we constructed an expression plasmid, in which the *mazF*ne1 gene was under the control of an IPTG-inducible promoter, and examined whether MazFne1 overexpression retarded *E. coli* growth. As shown in **Supplementary Figure 2B**, MazFne1 induction led to stable growth arrest, indicating that *mazF*ne1 indeed codes for a toxin. Taken together, these results suggest that MazEFne1 constitutes a TA system.

### MazFne1 Forms a TA System With Its Cognate Antitoxin MazEne1

**FIGURE 1:**
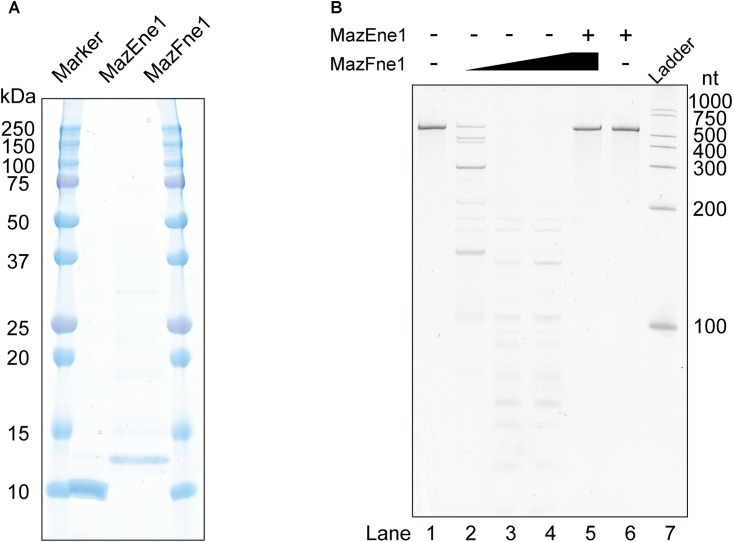
MazEF homologue isolated from *N. europaea* and their enzymatic activities. **(A)** Molecular weight and purity of MazEne1 and MazFne1. **(B)** A 533-nt artificially designed RNA (RNA 500-2) was incubated with MazEne1 and/or MazFne1: lane 1, a control reaction in the absence of enzymes; lanes 2–4, 1, 3, and 10 pmol of MazFne1 was added, respectively; lane 5, 10 pmol of MazFne1 was pre-incubated with 50 pmol of MazEne1 and RNA 500-2 was subsequently added; lane 6, 50 pmol of MazEne1 was added; lane 7, ladder.

To evaluate whether MazEFne1 functions as a TA system at the molecular level, we purified these enzymes using affinity chromatography (**Figure 1A**). As shown in **Figure 1B**, dose-dependent RNA degradation was detected following treatment of the RNA substrate with MazFne1, suggesting that MazFne1 constitutes an endoribonuclease (**Figure 1B**, lanes 2–4). Notably, the substrate remained intact when MazEne1 was added prior to RNA exposure (**Figure 1B**, lane 5); thus, the observed RNA cleavage was catalysed by MazFne1 but not by contaminating RNases. From these results, we concluded that the *mazEF*ne1 locus encodes a functional TA system.

### Modified RNA-Seq Indicates That UGG Is Necessary for MazFne1 Cleavage

**FIGURE 2:**
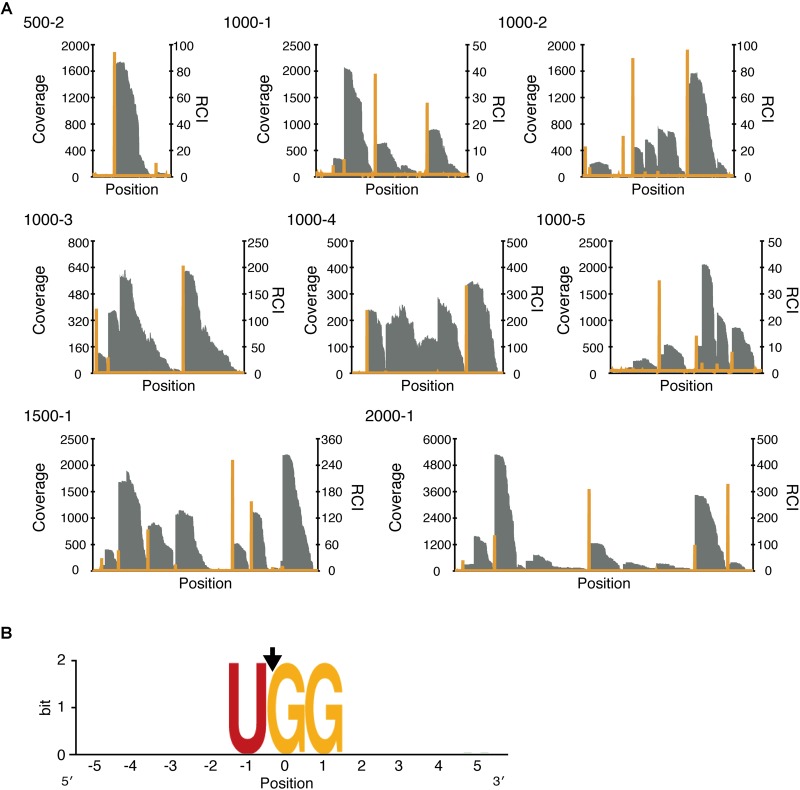
Analysis of the cleavage sequence of MazFne1. **(A)** Coverage (grey bar) and relative coverage increase (RCI; orange line), which is the value defined as the ratio of coverage at the (*n* + 1)th position to coverage at the *n*th position. **(B)** Nucleotide appearance frequency was visualised using WebLogo (Crooks et al., 2004). The black arrow indicates the position of the cleavage site.

Having confirmed the endoribonuclease activity of MazFne1, we proceeded to define its cleavage-specificity. Toward this end, we utilised a specialised RNA-Seq that we have recently developed (Miyamoto et al., 2016a). In this approach, we digested eight artificially designed RNAs containing diverse 500–2000 nt sequences (500-2, 1000-1, 1000-2, 1000-3, 1000-4, 1000-5, 1500-1, and 2000-1) with MazFne1, and analysed the 5′-end sequences of the fragmented RNAs. As MazF digests RNA at specific sequence motifs, reads carrying particular sequences at their 5′ boundaries, which match the MazF-cleaved sites, are enriched. Accordingly, the coverage tends to increase at corresponding nucleotide positions (**Figure 2A**; Miyamoto et al., 2016a).

To estimate the sequence determinants for MazFne1 cleavage, we extracted the sequences five bases up- and downstream of the identified nucleotide positions. After aligning the overall top 50 sequences, we found a strong consensus motif, UGG, from -1 to 1 positions (where the nucleotide position with coverage increase was numbered as zero; **Figure 2B, Supplementary Table 2**), suggesting that MazFne1 requires UGG triplets for its endoribonuclease activity and cut RNAs between U and the first G.

### UGG Is the Target of MazFne1

To further determine the sequence requirements of MazFne1, we adopted a fluorescence quenching technique and prepared oligonucleotides (listed in **Table 1**) that were modified with 6-carboxyfluorescein (6-FAM) on the 5′-end and black hole quencher-1 (BHQ-1) on the 3′-end. Because these 2 dyes are tethered by nucleotides and are in close vicinity, the fluorescence of 6-FAM is dampened. As these oligonucleotides are degraded, however, unquenched 6-FAM molecules accumulate, resulting in an increase in fluorescence intensity (Wang and Hergenrother, 2007).

**Table 1:** Fluorogenic oligonucleotides used in this study.

Name	Sequence (5′–3′)^a^
DR-13-UGG	AAAAAUGGAAAAA
D-13-AAA	AAAAAAAAAAAAA
R-13-UCUCG	UCUCGGUGCGUUG
R-13-UGACA	UGACACGAACCGC
R-13-GUUGU	GUUGUCAUGCCGG
DR-13-CGG	AAAAACGGAAAAA
DR-13-UGA	AAAAAUGAAAAAA
DR-13-UGU	AAAAAUGUAAAAA
DR-13-UGC	AAAAAUGCAAAAA


*^a^Underlined letters represent RNA nucleotides, whereas other letters represent DNA nucleotides.*

**FIGURE 3:**
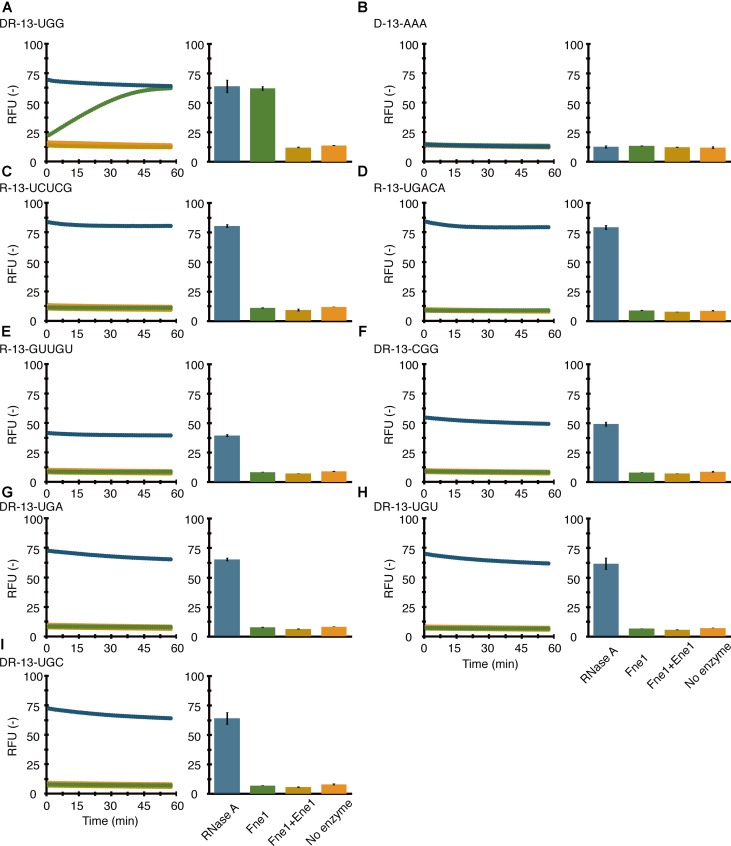
Sequence-specific RNA cleavage with MazFne1; 0.7 pmol of MazFne1 (green) or MazFne1 pre-incubated with 3.5 pmol of MazEne1 (yellow) was incubated with 20 pmol of fluorogenic oligonucleotides: **(A)** DR-13-UGG, **(B)** D-13-AAA, **(C)** R-13-UCUCG, **(D)** R-13-UGACA, **(E)** R-13-GUUGU, **(F)** DR-13-CGG, **(G)** DR-13-UGA, **(H)** DR-13-UGU, or **(I)** DR-13-UGC. In the control reactions, fluorescence intensities in the presence of 100 ng of RNase A (blue) and absence of enzymes (orange) at each time point (left) and the end point (right) were measured.

We sought to determine the MazFne1 target by monitoring the signal from 6-FAM. To corroborate the RNA-Seq result, we firstly utilised DR-13-UGG, which is a DNA-RNA chimeric oligonucleotide containing a UGG triplet in the middle of adenine repeats. As we anticipated, the oligonucleotide was sensitive to MazFne1 (**Figure 3A**, green plot). The cleavage was specifically caused by MazFne1 and was considered to occur at RNA nucleotide sites, because (i) cleavage activity was completely blocked upon MazEne1 addition (**Figure 3A**, yellow plot), and (ii) no detectable change in fluorescence intensity was observed when D-13-AAA, a DNA oligonucleotide, was used instead as the substrate for MazFne1 (**Figure 3B**).

We next prepared three additional RNA oligonucleotides (R-13-UCUCG, R-13-UGACA, and R-13-GUUGU), whose sequences are identical to that of RNA 1000-4 (one of the substrates used in the modified RNA-Seq) but lack the UGG motif. As expected, these RNAs were also tolerant to MazFne1 (**Figures 3C–E**). The results were concordant with the result from the modified RNA-Seq, suggesting that UGG is the target of MazFne1.

Lastly, we synthesised four DNA-RNA chimeric oligonucleotides (DR-13-CGG, DR-13-UGA, DR-13-UGU, and DR-13-UGC) to investigate whether MazFne1 cleaves 3-base motifs similar to UGG. As shown in **Figures 3F–I**, none of these oligonucleotides were degraded. Taken together, our findings demonstrated that MazFne1 constitutes an endoribonuclease that specifically cleaves the UGG triplet.

### The UGG Motif Is Abundant in Genes That Are Unique in the Ammonia-Oxidising Bacteria

**FIGURE 4:**
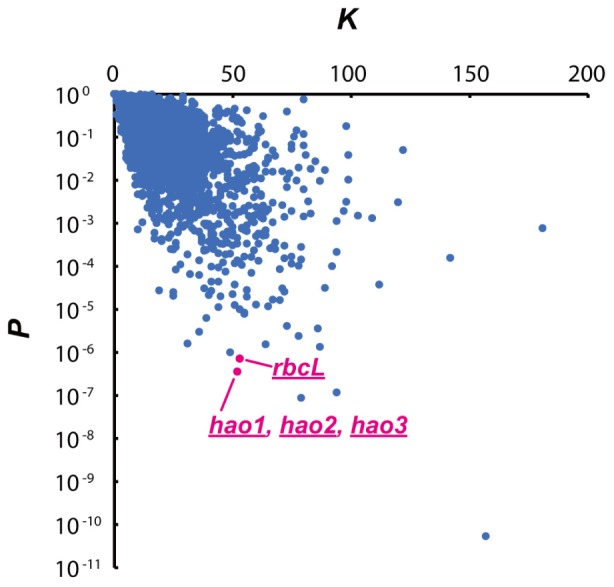
Relationship between the actual number of TGG triplets (*K*) and *P-*values in each CDS. A very small *P-*value indicates that the CDS is preferentially degraded by MazFne. Dots correspond to *hao*, and *rbcL* transcripts are highlighted.

The number of cleavage sequences in a transcript has been shown to correlate with mRNA stabilities (Zhu et al., 2009; Schifano et al., 2014). To evaluate the potential effect of MazFne1 on *N. europaea* cells, we performed statistical analysis and searched for genes containing multiple TGG sequences (Zhu et al., 2009) among the 2,646 coding sequences (CDS) identified in this organism. Specifically, we counted the actual number of TGG triplets in each CDS (*K*) and calculated the probabilities of each transcript containing *K* times or more TGG motifs (*P*), on the basis of nucleotide contents and the length of each CDS (**Figure 4**). Notably, when we extracted the top 10 CDS with the smallest *P-*values, the *hao* and *rbcL* genes, which are indispensable for ammonia oxidation and CO_2_ fixation, respectively, were detected (**Table 2**). As the CDS showing small *P*-values are considered to be preferentially digested, MazFne1 may serve to efficiently repress *N. europaea* cellular activities (see Discussion).

**Table 2:** Predicted prime targets of MazFne1.

Rank	Location	Gene symbol	Gene product	Length (bp)	Expected (*E*)^a^	Actual (*K*)^b^	*P*^c^
1	c199041-189847	–	Type I secretion C-terminal target domain containing protein	9195	89.43	157	5.30E-11
2	1783631–1786309	–	Dolichyl-phosphate beta-D-mannosyltransferase	2679	41.25	79	8.75E-08
3	c436078-432929	*hsdR*	Type I restriction endonuclease subunit R	3150	52.46	94	1.16E-07
4	c1048744-1047032	*hao2*	Hydroxylamine_oxidoreductase	1713	23.91	52	3.56E-07
5	c2215518-2213806	*hao1*	Hydroxylamine_oxidoreductase	1713	23.91	52	3.56E-07
6	c2540363-2538651	*hao3*	Hydroxylamine_oxidoreductase	1713	23.91	52	3.56E-07
7	c2076037-2074616	*rbcL*	Ribulose bisphosphate carboxylase large chain	1422	25.22	53	7.15E-07
8	c1601334-1600138	–	Aminotransferase	1197	22.82	49	9.90E-07
9	1894644-1898096	–	Antibiotic resistance protein VanZ	3453	50.25	87	1.33E-06
10	c988550-986691	*ftsH*	ATP-dependent metallopeptidase FtsH/Yme1/Tma family protein	1860	33.60	64	1.54E-06


*^a^E represents the expected motif count. ^b^K represents the actual motif count. ^c^P represents the probability of each transcript containing K times or more TGG motifs. A very small P-value indicates that the CDS may be the prime target of MazFne1.*

## Discussion

Microbes have to cope with sudden environmental alterations. A propitious strategy to survive in fluctuating surroundings is to regulate growth, with prokaryotic TA systems being a well-known example of such a strategy (Hall et al., 2017). In the present study, we characterised one of these representative TA systems, the MazEF family, encoded on the *N. europaea* chromosome. We found that MazEFne1 shares typical features of type II TA pairs: first, *mazE*ne1 and *mazF*ne1 are co-transcribed in *N. europaea* (**Supplementary Figure 2A**); second, ectopic expression of MazFne1 is toxic to bacterial cells (**Supplementary Figure 2B**); third, MazFne1 possesses endoribonuclease activity and MazEne1 counteracts its enzymatic activity (**Figure 1**); and fourth, MazFne1 specifically cleaves RNA at the UGG motif (**Figures 2, 3**).

Five MazF family members are encoded in the *N. europaea* chromosome. Although the sequence-specificities of these enzymes are not completely elucidated, they are, similar to other MazF homologues, considered to have different targets, triggers, and roles (Sat et al., 2003; Hazan et al., 2004; Tiwari et al., 2015; Gupta et al., 2017; Schifano and Woychik, 2017), because of their low sequence similarities and distinct loci. Indeed, we have previously shown that the *N. europaea* MazF encoded at ALW85_RS06130 (MazFne3; **Supplementary Figure 1A**) is an AAU-specific cutter. To the best of our knowledge, no stresses that activate MazFne1 have been identified. Further studies are needed to understand how these enzymes are beneficial to environmental fluctuations.

*N. europaea* production of reductant and energy via ammonia oxidation has been well elucidated. The process comprises a sequential oxidation reaction of ammonia to hydroxylamine (NH_3_ to NH_2_OH) and hydroxylamine to nitrite (NH_2_OH to NO_2_^-^); the former is catalysed by ammonia monooxygenase (AMO) and the latter by hydroxylamine oxidoreductase (recently designated as hydroxylamine dehydrogenase) (HAO) and putatively by nitric oxide oxidase (Caranto and Lancaster, 2017). Four electrons are released through hydroxylamine oxidation, half of which are used for ammonia oxidation and the other half for proton gradient formation, followed by ATP and NADH production (Arp et al., 2002; Arp and Stein, 2003). In the present study, we found that TGG triplets are strikingly abundant in the three copies of the *hao* genes (**Table 2**). Notably, these triplets are also relatively rich in *amo* genes (**Table 3**). Conceivably, the AMO and HAO transcripts are destabilised when MazFne1 is activated in *N. europaea* cells, thereby inhibiting cellular activities requiring energy and reductant.

**Table 3:** Actual and expected numbers of TGG motifs in *amo* genes^a^.

Gene symbol	Gene product	Length (bp)	Expected (*E*)	Actual (*K*)	*P*
amoC1/C2/C3	Ammonia monooxygenase, subunit C1/C2/C3	816/816/825	17.69/18.34/15.18	39/38/36	6.24E-06/3.09E-05/2.98E-06
amoA1/A2	Ammonia monooxygenase, subunit A1/A2	825/825	17.90/17.90	33/33	7.59E-04/7.59E-04
amoB1/B2	Ammonia monooxygenase, subunit B1/B2	1263/1263	21.48/21.48	41/41	9.85E-05/9.85E-05


*^a^E, K, and P represent values identical to those shown in **Table 2**.*

Moreover, in addition to ammonia oxidation, carbon fixation would also likely be impaired. *N. europaea* utilises the Calvon-Benson-Bassham (CBB) cycle to fix carbon dioxide (Chain et al., 2003), with this step being initiated by a type I ribulose 1,5-bisphosphate carboxylase/oxygenase (RubisCo), which is composed of a large subunit and small subunits (Wei et al., 2004). In our analysis, the *rbcL* gene, encoding the large subunit of RubisCo, is predicted to be sensitive to MazFne1 (**Table 2**). Taken together with the fact that in *N. europaea*, the CBB cycle is powered by ATP and NADH generated from ammonia oxidation (Chain et al., 2003), MazFne1 would supposedly severely compromise the biosynthesis processes of this bacterium.

It has been previously observed that type II TA systems are abundant in some slowly growing bacteria, including *N. europaea* (Pandey and Gerdes, 2005). Although it remains unclear why these seemingly redundant TA pairs exist in this bacterium, the number of TA pairs might be associated with the growth rate. Indeed, 99.9% of *N. europaea* coding sequences contain at least one TGG motif. Presumably, MazFne1 arrests *N. europaea* growth by directly shutting down almost all protein synthesis, in addition to the indirect downregulation of many biochemical reactions through the inhibition of ammonia oxidation and carbon fixation. Given that a low metabolic state represents a common bacterial strategy to improve stress resistance and that TA systems are implicated in the formation of such states, referred to as “dormant and/or viable but non-culturable” (Helaine et al., 2014; Li et al., 2014; Ayrapetyan et al., 2015; Tiwari et al., 2015), *N. europaea* might withstand environmental stresses with the aid of MazFne1.

In summary, we found that MazFne1 codes for a functional endonucleolytic toxin and constitutes a TA pair with its cognate antitoxin, MazEne1. Additionally, MazFne1 digests RNAs at UGG sites in a ribosome-independent manner. Our finding indicates that *N. europaea* might benefit from the growth modulation mediated by MazFne1 under stressful conditions.

## Author Contributions

TM, AY, YO, MT, RA, ST, and NN conceived and designed the experiments. TM, AY, YO, and MT performed the experiments. TM, AY, YO, MT, and RA analyzed the data. TM wrote the paper.

## Conflict of Interest Statement

The authors declare that the research was conducted in the absence of any commercial or financial relationships that could be construed as a potential conflict of interest.
