# Corrigendum: *Nitrosomonas europaea* MazF Specifically Recognises the UGG Motif and Promotes Selective RNA Degradation

**DOI:** 10.3389/fmicb.2019.00634

**Published:** 2019-04-03

**Authors:** Tatsuki Miyamoto, Akiko Yokota, Yuri Ota, Masako Tsuruga, Rie Aoi, Satoshi Tsuneda, Naohiro Noda

**Affiliations:** ^1^Department of Life Science and Medical Bioscience, Waseda University, Tokyo, Japan; ^2^Biomedical Research Institute, National Institute of Advanced Industrial Science and Technology (AIST), Ibaraki, Japan

**Keywords:** toxin-antitoxin system, MazEF, sequence-specificity, RNase, *Nitrosomonas europaea*, ammonia oxidation, carbon fixation

In the original article, there was a mistake in Figure 4 as published. Figure 4 shows the relationship between the actual number of TGG triplets (*K*) and *P*-values in each CDS. In this figure, we mistook *hao* for *rbcL*; the dot which represents *hao* corresponds to the *rbcL* transcript, while the dot which represents *rbcL* corresponds to the *hao* transcripts. The corrected Figure 4 appears below.

**Figure 4:**
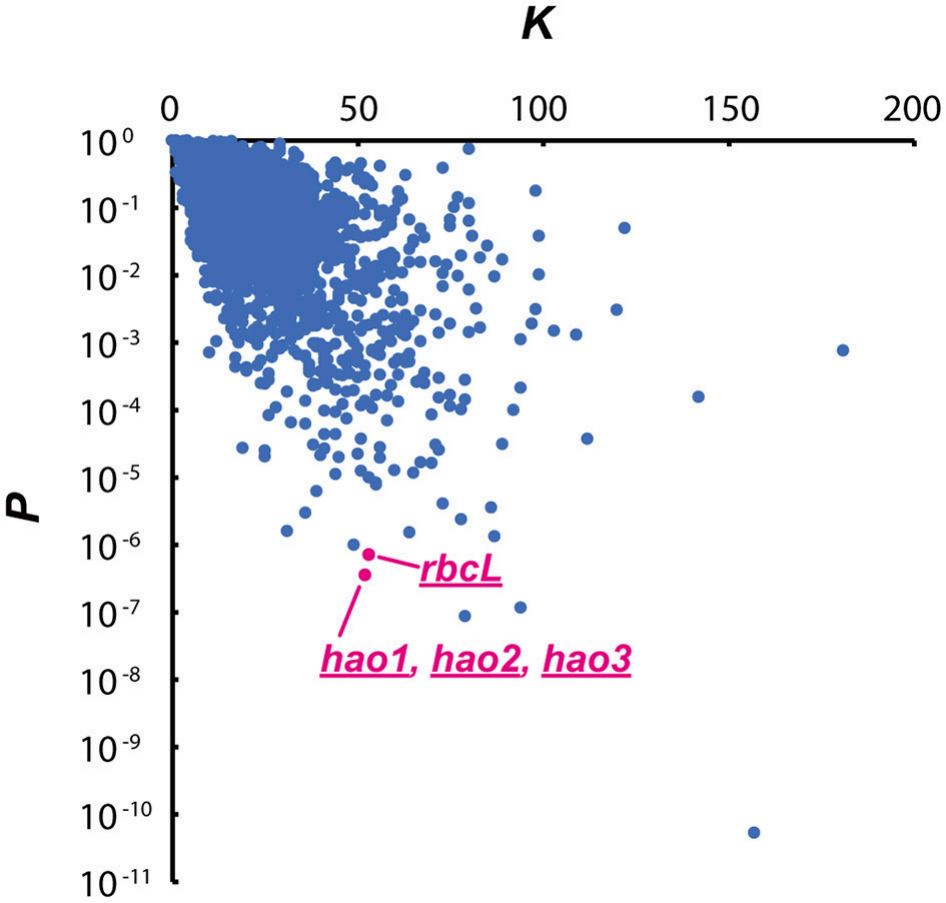
Relationship between the actual number of TGG triplets (*K*) and *P-*values in each CDS. A very small *P-*value indicates that the CDS is preferentially degraded by MazFne. Dots correspond to *hao*, and *rbcL* transcripts are highlighted.

The authors apologize for this error and state that this does not change the scientific conclusions of the article in any way. The original article has been updated.

