# Supplementary material for: Nitrosomonas europaea MazF Specifically Recognises the UGG Motif and Promotes Selective RNA Degradation
Source: Front Microbiol. 2018 Oct 8;9:2386. doi: 10.3389/fmicb.2018.02386 (PMC6186784; doi:10.3389/fmicb.2018.02386)
Supplement: Supplementary file 1 [file Data_Sheet_1.docx]

Supplementary Material

*Nitrosomonas europaea* MazF specifically recognises the UGG motif and promotes selective RNA degradation

Tatsuki Miyamoto^†^, Akiko Yokota^†^, Yuri Ota, Masako Tsuruga, Rie Aoi, Satoshi Tsuneda^*^, and Naohiro Noda^*^

^†^These authors have contributed equally to this work.

*** Correspondence:**Dr. Satoshi Tsuneda
[stsuneda@waseda.jp](mailto:stsuneda@waseda.jp)
Dr. Naohiro Noda
[noda-naohiro@aist.go.jp](file:///D:\CF-SX2_Users_Akiko\Documents\2014年4月-Bioanalytical%20RG\執筆論文\MazF-NE1\20180717_Frontiers_in_MicroBiol\noda-naohiro@aist.go.jp)


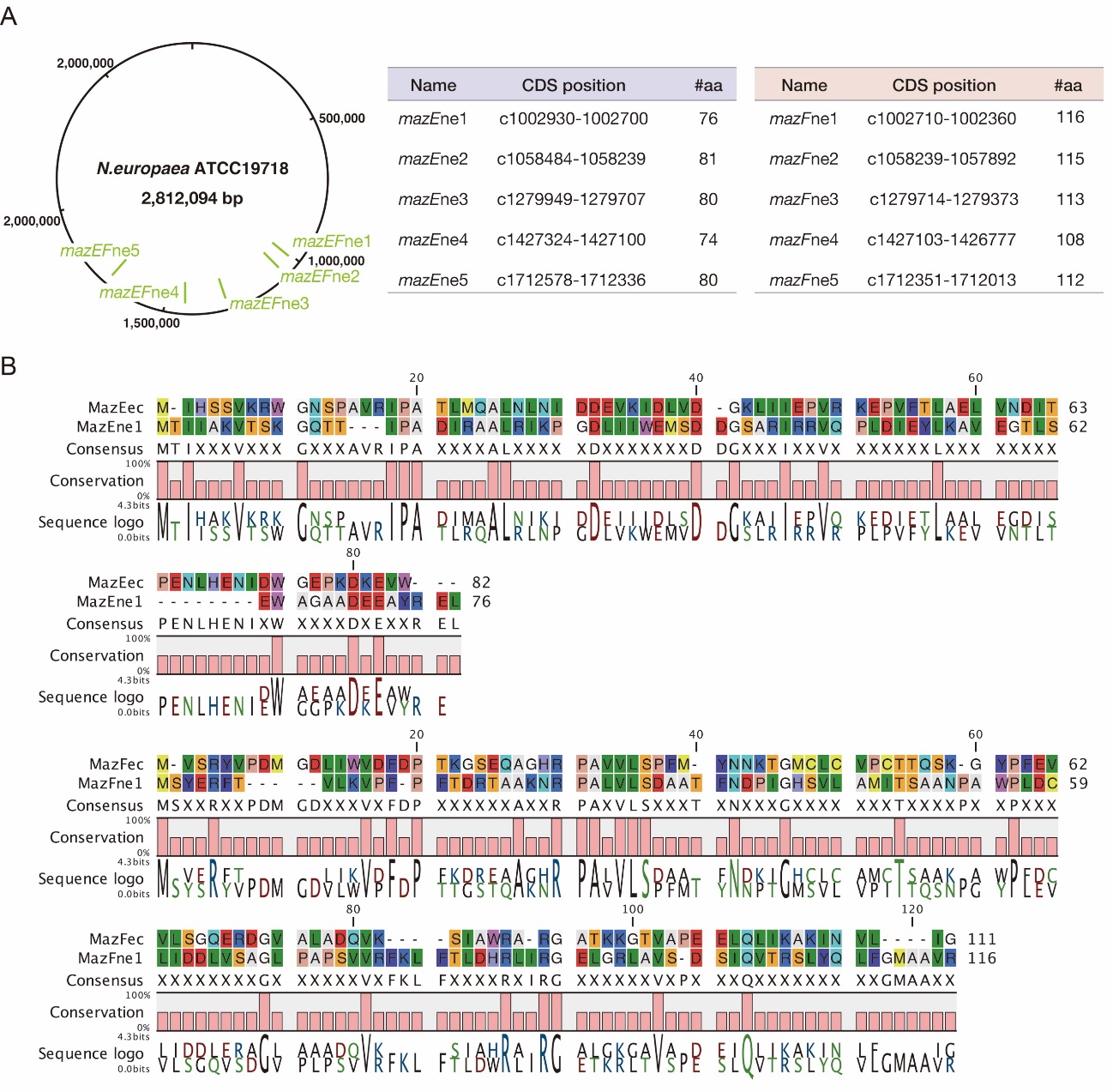


**Supplementary Figure S1** *N. euroaea* MazEF pairs (A) Five MazEF loci predicted in *N. europaea*. (B) Pairwise alignment of *E. coli* MazEF (MazEec and MazFec) and *N.europaea* MazEF (MazEne1 and MazFne1); upper panel, MazE comparison; lower panel, MazF comparison.


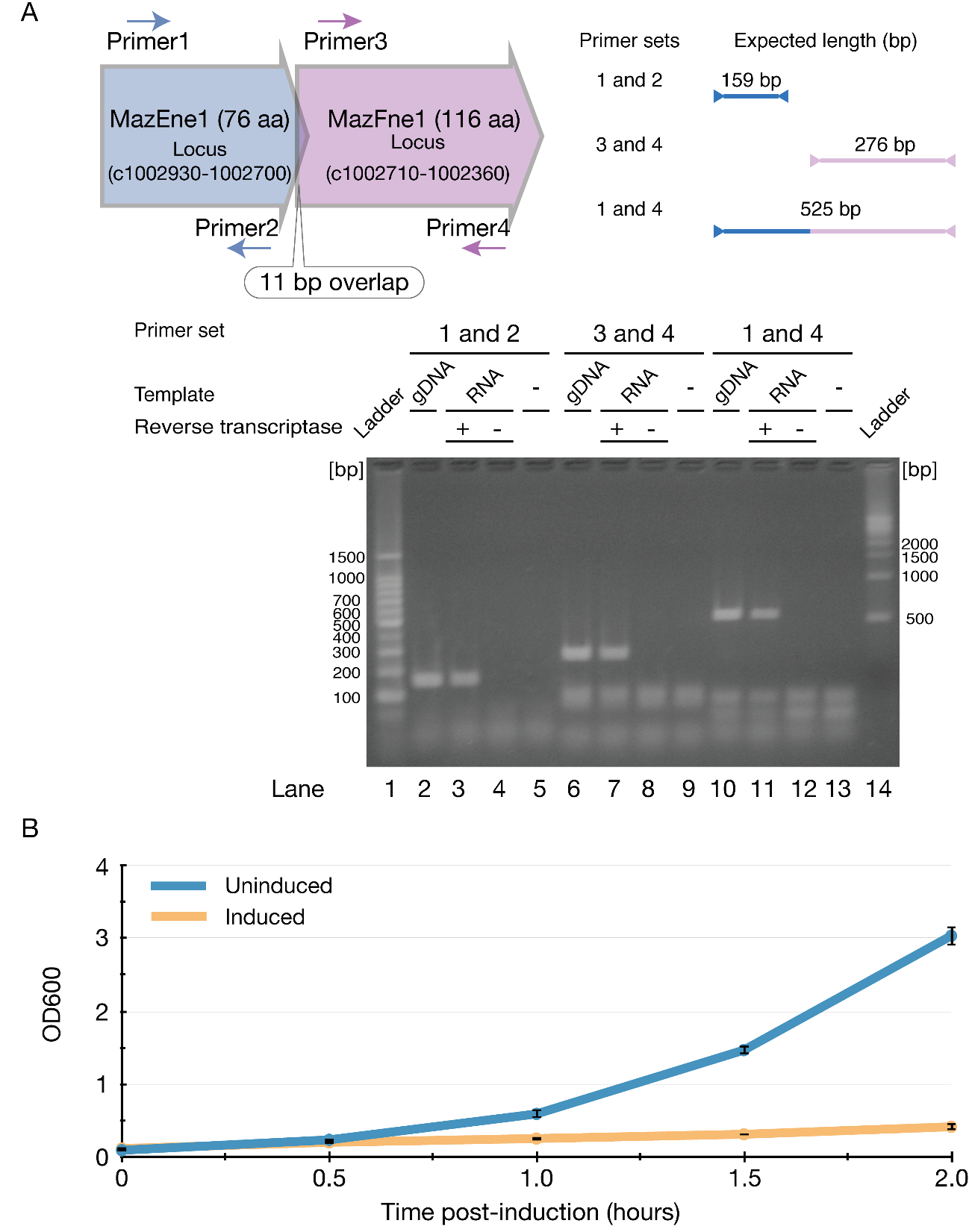


**Supplementary Figure S2** The *mazEF*ne1 pair in *N. europaea* (A) Schematic representation of *mazEF*ne1 genes and the primer sets used in this study (top) and the electrophoresed bands (bottom): lanes 1 and 14, DNA ladders; lanes 2, 6, and 10, genomic DNA of *N. europaea* was used as a template; lanes 3, 7, and 11, cDNA was used as a template; lanes 4, 8, 12, extracted RNA without reverse transcriptase added to exclude the possibility of genomic DNA contamination; lanes 5, 9, 13, no template addition. (B) Growth profile of *E. coli* cells with (orange) or without (blue) MazFne1 induction.

**Supplementary Table S1** PCR primers used in this study

| Name | Sequence (5' to 3') |
| --- | --- |
| *mazE*ne1 forward (Primer1) | CCTCAAAGGGACAGACCACG |
| *mazE*ne1 reverse (Primer2) | GCGTTCCCTCCACTGCTTTC |
| *mazF*ne1 forward (Primer3) | CGTACGGCTGCCAAAAACCG |
| *mazF*ne1 reverse (Primer4) | AAGCTGATACAGAGATCGTGTG |

**Supplementary Table S2** Top 50 sequences showing large RCI

| Rank | RNA type | Position | Coverage | RCI | Sequence (5′ to 3′) ^a^ | Rank | RNA type | Position | Coverage | RCI | Sequence (5′ to 3′) ^a^ |
| --- | --- | --- | --- | --- | --- | --- | --- | --- | --- | --- | --- |
| 1 | 1000-4 | 782 | 332 | 332 | ACUUUGGUUGC | 26 | 1000-5 | 828 | 853 | 8.12 | AUGGUGGUACA |
| 2 | 2000-1 | 1861 | 329 | 329 | AUAAUGGUAGG | 27 | 1000-2 | 51 | 192 | 7.38 | CGCAUGGUUCU |
| 3 | 2000-1 | 916 | 1239 | 309.8 | CUCAUGGCAAU | 28 | 1500-1 | 1279 | 194 | 6.93 | UAUGUGGUGCA |
| 4 | 1500-1 | 952 | 504 | 252 | AUAUUGGCAGA | 29 | 1000-1 | 190 | 2075 | 6.74 | UAAAUGGACAA |
| 5 | 1000-4 | 104 | 239 | 239 | UAGUUGGUUCA | 30 | 2000-1 | 131 | 1573 | 4.93 | AAUAUGGCUGA |
| 6 | 1000-3 | 616 | 610 | 203.3 | AACAUGGUAGA | 31 | 1000-2 | 516 | 769 | 4.5 | UAUAUGGGAGA |
| 7 | 1500-1 | 1080 | 1106 | 158 | GACGUGGAAGG | 32 | 1000-1 | 116 | 352 | 4.4 | GAACUGGGUUA |
| 8 | 2000-1 | 272 | 5278 | 135.3 | UCAUUGGCCCA | 33 | 1000-5 | 825 | 105 | 4.38 | GCUAUGGUGGU |
| 9 | 1000-3 | 24 | 122 | 122 | CGAGUGGACGC | 34 | 2000-1 | 1147 | 328 | 4.32 | CAACUGGGACC |
| 10 | 2000-1 | 1636 | 3451 | 98.6 | CCCGUGGACCA | 35 | 2000-1 | 485 | 465 | 4.31 | ACCCUGGCGCU |
| 11 | 1000-2 | 717 | 1250 | 96.15 | UAACUGGCCUG | 36 | 1000-2 | 429 | 568 | 4.09 | CGUCUGGCUUA |
| 12 | 1500-1 | 375 | 850 | 94.44 | AGGUUGGAAUC | 37 | 1000-5 | 623 | 2057 | 4 | CAUCUGGACGU |
| 13 | 500-2 | 148 | 1698 | 94.33 | AUACUGGAGAA | 38 | 1000-5 | 726 | 1152 | 3.63 | CGCUUGGAUAA |
| 14 | 1000-2 | 345 | 449 | 89.8 | CUCGUGGAAGC | 39 | 1500-1 | 82 | 403 | 3.57 | GCCAUGGUUCC |
| 15 | 1500-1 | 174 | 1683 | 46.75 | CUGAUGGGAUU | 40 | 2000-1 | 1373 | 359 | 2.87 | AAUGUGGGUAG |
| 16 | 2000-1 | 56 | 283 | 40.43 | UAGGUGGUAAG | 41 | 1000-4 | 586 | 287 | 2.39 | UUAGUGGGCGG |
| 17 | 1000-1 | 403 | 312 | 39 | UUGAUGGUGGC | 42 | 1000-3 | 184 | 597 | 2.35 | AAUUUGGAGGU |
| 18 | 1000-5 | 332 | 351 | 35.1 | UAUCUGGAAUA | 43 | 1000-2 | 922 | 279 | 2.1 | CAAAUGGCGCA |
| 19 | 1000-3 | 104 | 362 | 30.17 | CCAAUGGAUGU | 44 | 1000-5 | 153 | 229 | 2.1 | CGGUUGGUCAC |
| 20 | 1500-1 | 60 | 116 | 29 | GUUAUGGACCA | 45 | 1000-1 | 406 | 619 | 1.98 | AUGGUGGCAGA |
| 21 | 1000-1 | 757 | 870 | 28.06 | GCUUUGGUAGA | 46 | 1000-5 | 111 | 104 | 1.79 | ACAGUGGUUCG |
| 22 | 1500-1 | 563 | 1068 | 14.83 | GAGGUGGAGCG | 47 | 2000-1 | 535 | 741 | 1.66 | ACGUUGGGUCG |
| 23 | 1000-5 | 585 | 525 | 14.19 | UUAGUGGCCCA | 48 | 1000-1 | 578 | 210 | 1.56 | UACCUGGUGUC |
| 24 | 1500-1 | 1293 | 2186 | 11.33 | UUUCUGGCCAA | 49 | 2000-1 | 430 | 288 | 1.55 | UACAUGGUAAC |
| 25 | 1000-4 | 232 | 185 | 9.74 | GCUUUGGUUGU | 50 | 1000-5 | 364 | 533 | 1.47 | CGAAUGGGCCG |

a Underlined letters represent the base with significant coverage increase
